# Pharmaceutical access in medical security systems and health policy mobilization: cross-national evidence on TRIPS compulsory licensing from 2001 to 2024

**DOI:** 10.3389/fpubh.2026.1944235

**Published:** 2026-10-01

**Authors:** Dongmei Wang

**Affiliations:** The Institute of Law, Taiwan Research Institute, Xiamen University, Xiamen, China

**Keywords:** ART coverage, cross-national public panel data, global health governance, health policy mobilization, medical security systems, pharmaceutical access, public health governance, TRIPS compulsory licensing

## Abstract

**Background:**

Pharmaceutical access is essential for medical security systems to convert health protection commitments into actual treatment coverage. TRIPS compulsory licensing gives WTO members legal space to limit patent exclusivity under specific conditions, but legal authorization does not automatically become health policy mobilization. Existing research has not sufficiently tested the public health pressure and governance resource conditions under which compulsory licensing is mobilized in a WTO-corrected country-year risk set.

**Methods:**

Drawing on the Medicines Law & Policy TRIPS Flexibilities Database, UNAIDS, the Global Fund, the World Bank, and WHO, this study constructs a country-year panel of 103 WTO members from 2001 to 2024, with 2,276 observations. Two-way fixed effects linear probability models are used as the main analysis of the institutional mobilization conditions of compulsory licensing-related events. GEE, first-event, and Poisson models are used as robustness checks, while HIV/AIDS-related compulsory licensing models and ART coverage outcome models are used as supplementary boundary analyses. ART coverage is treated only as an indicator of HIV/AIDS treatment access, not as a general measure of access to medicines.

**Results:**

Lower ART coverage is relatively consistently and negatively associated with compulsory licensing events, and supplementary analyses of HIV/AIDS-related compulsory licensing support the same direction. Observed Global Fund HIV assistance is positively associated with compulsory licensing under the all-event measure and in several robustness models, suggesting that governance-resource connections may be associated with the feasibility of converting legal flexibilities into policy action. AIDS deaths, TB incidence, GDP per capita, health expenditure, out-of-pocket expenditure, and manufactured exports do not show stable main effects across models. Supplementary outcome-boundary models do not support the claim that HIV/AIDS-related compulsory licensing significantly increases ART coverage.

**Discussion:**

TRIPS compulsory licensing is better understood as a health policy instrument addressing pharmaceutical access risks within medical security systems. In the current observational panel, its mobilization is associated with HIV/AIDS treatment access pressure and observed governance-resource connections, rather than operating as a medicine access improvement mechanism that current cross-national data can directly establish.

## 1 Introduction

Pharmaceutical access is a core component of medical security systems and global public health governance. The supply of safe, effective, and affordable medicines determines whether health policy commitments can be translated into treatment coverage, and it also depends on the institutional capacity linking medicine payment, procurement and supply, and public health governance. Patent exclusivity supports innovation incentives, but in infectious disease treatment and low-resource settings, it may intensify price barriers, restrict the entry of generic medicines, and narrow the space for treatment coverage (1–4). This tension is especially pronounced in the treatment of global infectious diseases such as HIV/AIDS, tuberculosis, and malaria, where patient populations in low- and middle-income countries often face a serious mismatch between patented drug prices and public health budgets. Article 31 and Article 31bis of the Agreement on Trade-Related Aspects of Intellectual Property Rights, TRIPS, together with the Doha Declaration, embed public health considerations within international intellectual property rules and provide members with legal space to limit patent exclusivity under specific conditions (2, 5).

On these issues, doctrinal legal research and case studies have identified the legality, low frequency of use, price negotiation function, and institutional barriers of compulsory licensing (4, 6–10). Some cross-national empirical studies have also advanced the empirical understanding of compulsory licensing from the perspectives of legislative adoption (11), temporal dynamics and institutional maturity (12), and political economy. These studies provide an important basis for this article by explaining compulsory licensing rules in terms of public health purposes, legality, and institutional function. However, the existence of a public health purpose does not necessarily mean that actual initiation is concentrated in country-year contexts with greater public health pressure, nor does use necessarily translate into observable health outcomes. It is therefore necessary to distinguish and test two questions: under what conditions compulsory licensing is actually mobilized, and whether that mobilization appears in cross-national data as a change in health outcomes.

To address this gap, this study shifts the focus from the theoretical legal availability of compulsory licensing and its threat function after invocation to the actual conditions of compulsory licensing mobilization and its observable public health associations. Specifically, it places compulsory licensing-related events within a country-year risk set covering 103 WTO members. It then examines whether treatment access pressure, disease burden, health system absorptive conditions, and connections to global health governance resources are stably associated with the occurrence of compulsory licensing events. This framing connects TRIPS compulsory licensing to a public health policy question: whether legal exceptions can be converted into pharmaceutical access governance through medical security systems, procurement arrangements, and transnational health-resource connections. Because ART coverage reflects only pressure in HIV/AIDS treatment access, it cannot represent all public health pressure or all dimensions of pharmaceutical access. This study therefore distinguishes all compulsory licensing events from HIV/AIDS-related compulsory licensing events and tests whether the relationship remains after disease-area matching. On this basis, the study separates institutional mobilization from health outcome conversion and asks whether the current observational data support a causal interpretation linking compulsory licensing use to improved ART coverage.

## 2 Literature review

### 2.1 TRIPS public health exceptions and policy space in international economic law

TRIPS public health exceptions are not an external negation of the patent system, but institutional flexibilities reserved within international intellectual property rules for public health objectives (2, 3). While establishing obligations for intellectual property protection, TRIPS provides members with lawful pathways to limit patent exclusivity in public health crises through Article 31, the Doha Declaration, and Article 31bis (2, 13). This design expresses a balance: members must comply with minimum standards of intellectual property protection, but they retain flexible policy space under specific conditions (3). Article 31 mainly concerns domestic markets and government use, allowing members to issue compulsory licenses after meeting specified procedural requirements (2, 5). Article 31bis seeks to create a cross-border supply route for members lacking pharmaceutical manufacturing capacity, while introducing additional requirements concerning import verification, export limitations, notification obligations, and procurement coordination (5, 14). Although these requirements are intended to prevent patent abuse and drug diversion, they objectively increase the institutional cost of this route and raise the threshold for mobilization by members without manufacturing capacity (14).

Research on the domestic incorporation of TRIPS public health exceptions further shows that the institutional basis for compulsory licensing has been formed within many members. Most WTO members have incorporated compulsory licensing provisions into domestic patent law, indicating that TRIPS flexibilities exist not only in international legal texts but also as domestic legal bases for activation (15). At the same time, the interpretive space of TRIPS public health exceptions allows members to retain different degrees of policy flexibility in public health, government use, emergencies, and non-commercial use (11, 16). Yet a contrast remains between institutionalization and infrequent actual use. This contrast is the immediate starting point for this study’s examination of the conditions under which compulsory licensing is actually mobilized.

### 2.2 Compulsory licensing practice, low use, and institutional implementation barriers

Practice after the Doha Declaration further magnifies this contrast. Existing database studies and systematic reviews generally agree that compulsory licensing events are not evenly distributed among all members with legal authorization, but are concentrated in particular periods, countries, and disease areas (4, 6, 9). Compulsory licensing is therefore not a routine policy instrument that members naturally activate once they obtain legal authorization. It is closer to a low-frequency policy event mobilized under specific public health, market, and governance conditions. The contrast between institutionalization and infrequent actual use is thus not merely a doctrinal legal gap. It is also an empirical question for this study: under what conditions does legal space in international economic law become practically available and capable of actual mobilization by members?

Treaty authorization alone cannot generate medicine supply, fiscal payment, regulatory approval, procurement contracts, or political tolerance (14, 17). The practical availability of TRIPS public health exceptions depends on whether legal space can be converted into executable action through domestic institutions, market supply, and transnational governance arrangements. Existing research shows that this conversion process is constrained by multiple conditions. In the legal-procedural dimension, members must complete domestic administrative initiation, patent validity assessment, and compensation arrangements. In the production and supply dimension, the approval and deployment cycle of pharmaceutical manufacturing networks may increase supply uncertainty, and local pharmaceutical production capacity depends on coordination between public health policy and industrial policy (18–20). In the political dimension, external trade pressure, reactions from patent holders, and international political relations may affect whether members initiate or implement compulsory licensing (21–23). In the procurement and organizational dimension, drug procurement channels, supply chain management, and market competition structures affect the practical executability of compulsory licensing as a policy instrument (4, 24). Even though Article 31bis provides a cross-border supply route for members without manufacturing capacity, its formal use remains extremely limited, further showing that procedural and supply constraints in the institutional conversion chain do not automatically disappear because legal authorization exists (5, 25). Together, these factors constitute bottlenecks in the conversion from legal space to policy action and show that the practical availability of compulsory licensing cannot be explained only by treaty authorization or the existence of domestic legislation.

Research on the time structure from drug launch to attempted compulsory licensing, institutional credibility, and regulatory threats also indicates that compulsory licensing is not an automatic event after treaty authorization. It is a strategic instrument shaped by market-entry conditions, institutional absorptive capacity, and the political economy environment (12, 26–28). This provides an analytical starting point for this study to examine initiation conditions from the perspectives of public health pressure, health system absorptive conditions, and connections to global health governance resources.

### 2.3 Clues to initiation conditions: public health pressure and global health governance resources

Drug market entry and price access problems can be partly expressed at the country-year level as treatment access pressure and disease burden. Institutional absorptive capacity can be observed indirectly through health system resources and general state capacity, while the political economy environment can be empirically characterized through connections to global health governance resources. On this basis, this study summarizes the institutional conversion bottlenecks identified by previous research into four empirical analytical clues: treatment access pressure, disease burden, health system absorptive conditions, and connections to global health governance resources.

Existing research on HIV/AIDS treatment, antiretroviral drug prices, and civil society action suggests that treatment access pressure and disease burden may be important conditions under which members consider mobilizing compulsory licensing. Experiences in Thailand and Brazil show that insufficient treatment coverage and pressure from drug prices directly motivated governments to initiate compulsory licensing (7, 8, 21, 22).

International assistance and global health governance networks may affect the feasibility of mobilizing compulsory licensing through financing, procurement procedures, project implementation, transparency, and accountability mechanisms. As the world’s largest financing platform for HIV/AIDS, tuberculosis, and malaria responses, the Global Fund provides not only fiscal resources but also governance links with recipient health systems through procurement management, supply chain coordination, performance assessment, and anti-corruption mechanisms (29–31). Studies of procurement and supply chain reform further show that pooled procurement and market-shaping mechanisms supported by the Global Fund can help reduce drug procurement costs and improve supply predictability (32–35). Global health governance resources may therefore affect whether members can convert legal space into executable policy action.

Finally, general state capacity and economic structure may also affect compulsory licensing mobilization. Economic development, health system resources, and production and supply capacity influence fiscal payments, administrative execution, drug procurement, and supply organization. However, these broad structural variables may not sufficiently explain the specific institutional absorptive capacity required to initiate compulsory licensing (19, 20, 24, 36).

### 2.4 Consequences of compulsory licensing use: access to medicines outcomes cannot be simply assumed

Compulsory licensing has a clear public health institutional purpose, but its public health consequences cannot be inferred directly from legal use itself. Existing research on ARV prices (10), experiences in Thailand and Brazil (7, 8), compulsory licensing requests in Ecuador, and global generic supply networks (37, 38) show that compulsory licensing may affect access to medicines through price negotiation, alternative supply, policy signaling, and market competition pressure. Yet these effects are highly dependent on specific market structures, supply sources, procurement implementation, and domestic policy coordination. There is therefore no automatic link between the institutional mobilization of compulsory licensing and health outcomes. Even if a member initiates or threatens compulsory licensing, the legal instrument may remain at the negotiation or authorization stage if executable procurement contracts, qualified suppliers, drug registration pathways, fiscal payment capacity, and health service delivery systems are absent. In that case, it may not stably translate into lower prices, improved supply, or expanded treatment coverage.

This point has important methodological implications for the present study. The relationship between compulsory licensing and public health may be asymmetric: the context of initiation is usually closely related to drug prices, treatment coverage, and public health crises, but use itself cannot be presumed to improve health outcomes. In other words, compulsory licensing is first an institutional mobilization instrument under public health pressure. Its public health significance lies in whether members can invoke policy space under international economic law. Whether that invocation can further translate into observable health outcomes depends on procurement, supply, fiscal, and health system conditions outside the legal instrument itself. Based on this understanding, research on compulsory licensing needs to distinguish two levels: first, the conditions under which the legal instrument is actually mobilized; second, whether such mobilization further translates into observable health outcomes. The first concerns the practical availability of policy space in international economic law, while the second concerns the public health conversion boundary of that policy space. Without this distinction, the public health purpose, institutional mobilization, and health outcome improvement of compulsory licensing are easily conflated.

In sum, existing research has advanced the understanding of compulsory licensing from treaty interpretation, domestic legislative adoption, practice trends, price outcomes, and institutional barriers, and cross-national empirical research already exists (11, 12, 26, 27). What remains relatively underdeveloped is simultaneous testing of two related but distinct questions within a country-year risk set corrected for WTO membership and accession year. The first is whether actual compulsory licensing mobilization is more likely in contexts of stronger treatment access pressure, disease burden, or connection to global health governance resources. The second is whether compulsory licensing use is further associated with observable health outcome changes. This study therefore asks the following research questions:

*RQ1*: In a country-year risk set of WTO members, are treatment access pressure and disease burden stably associated with the occurrence of compulsory licensing events?

*RQ2*: Is connection to global health governance resources, as reflected by Global Fund HIV assistance, stably associated with the occurrence of compulsory licensing events?

*RQ3*: Can conventional economic and capacity variables, such as GDP per capita, health expenditure, and the share of manufactured exports, stably explain compulsory licensing use?

*RQ4*: Do the current data support a causal interpretation linking compulsory licensing use to improved ART coverage?

## 3 Research design

### 3.1 Unit of analysis, data sources, and the WTO risk set

This study adopts a country-year panel design and treats compulsory licensing-related events as low-frequency policy events. It examines whether the public health pressure, health system absorptive conditions, and connections to global health governance resources faced by WTO members in different years are associated with compulsory licensing mobilization. The advantage of a panel design is that it can address both unobserved cross-country differences and common-year shocks. Compared with a cross-sectional design, the panel design can use country fixed effects to control for time-invariant institutional, cultural, and geographic factors. Compared with case comparison, it allows variable relationships to be tested for cross-national stability in a larger sample. This study focuses on observational associations and predictive relationships and does not claim strict causal identification.

This study uses five public data sources to construct the country-year panel. Data on compulsory licensing events come from the Medicines Law & Policy TRIPS Flexibilities Database, hereafter the MLP database, which systematically records global use of TRIPS flexibility instruments since 2001 (9, 39). ART coverage and AIDS-related variables come from UNAIDS AIDSinfo (40, 41). Global Fund HIV assistance comes from the Global Fund Data Service (42), which records annual country-level disbursements for HIV programs (29, 30, 43). Macro-level control variables, including GDP per capita, population, urbanization, health expenditure, out-of-pocket expenditure, and the share of manufactured exports, come from the World Bank World Development Indicators (44). TB incidence and mortality come from the WHO Global Tuberculosis Program (45).

The unit of analysis is a country-year risk set corrected for WTO membership and accession year. Because compulsory licensing is an institutional instrument under the TRIPS framework, members are meaningfully at institutional risk in the sense used here only after joining the WTO and becoming subject to TRIPS obligations. The original expanded sample contained 3,118 country-years for 129 low-income, lower-middle-income, and upper-middle-income countries. After verification, 26 countries or territories not confirmed in the official WTO member list were excluded, as were pre-accession years for 19 countries, and duplicate Tanzania ISO 3-year keys caused by Zanzibar mapping. The final risk set contains 2,276 post-accession country-years for 103 WTO members. Among these observations, 110 record at least one compulsory licensing-related event, and 88 record executed compulsory licensing events. A zero value in the risk set means that no compulsory licensing event was observed in the MLP database. It does not mean that an official authority has confirmed that no compulsory licensing occurred in that country-year.

### 3.2 Dependent variables: compulsory licensing events and a supplementary HIV/AIDS-related measure

This study operationalizes compulsory licensing use as observed country-year policy events in the MLP database and defines three main dependent variables. First, any compulsory licensing event, cl_any_binary, indicates whether any type of compulsory licensing-related event occurred in a country-year, including execution, threat, application, and pending status, and takes the value 0 or 1. Second, executed compulsory licensing event, cl_executed_binary, indicates whether an actually executed compulsory licensing event occurred in a country-year and takes the value 0 or 1. Third, compulsory licensing event count, flex_count, records the total number of compulsory licensing-related events in a country-year. These three dependent variables capture whether compulsory licensing occurred, whether it was actually executed, and the density of events. Any compulsory licensing event captures broad institutional mobilization and does not mean that a compulsory license was actually executed. Executed compulsory licensing captures actual use in a stricter sense. The main analysis uses any compulsory licensing-related event as the principal measure and uses executed compulsory licensing to test whether the results remain under a stricter actual-use measure.

The main analysis uses all compulsory licensing events to examine the overall institutional mobilization conditions of TRIPS compulsory licensing and related flexibility instruments. In other words, the all-event models ask in what country-year contexts members are more likely to mobilize this legal instrument. They do not test treatment outcomes for pharmaceutical policy in a specific disease area. Because ART coverage corresponds only to HIV/AIDS treatment and cannot represent the disease area of all compulsory licensing events, this study further constructs HIV/AIDS-related compulsory licensing events as a supplementary measure. Under the strict measure, events in which the diseases field is marked as HIV/AIDS, HIV/AIDS +, HIV, or HIV/AIDS, HBV are identified as HIV/AIDS-related compulsory licensing events. Nirmatrelvir/ritonavir events in the Covid-19 context and non-ART drug events are not included. Under this measure, the MLP event table contains 114 HIV/AIDS-related compulsory licensing events. After entering the WTO risk set, there are 95 events involving 83 country-years and 52 countries. This supplementary measure is used to test whether the relationship between ART coverage and compulsory licensing mobilization remains after disease-area matching.

### 3.3 Core explanatory variables and control variables

This study distinguishes public health pressure into treatment access pressure and disease burden. ART coverage, art_coverage, is the core treatment access pressure variable and measures the coverage gap in access to antiretroviral therapy among people living with HIV/AIDS. It refers to the share of people living with HIV who receive antiretroviral therapy among all people living with HIV who need treatment, based on UNAIDS estimates (40). Lower ART coverage indicates a larger HIV/AIDS treatment coverage gap (46, 47). ART coverage is not treated in this study as the outcome variable of all compulsory licensing events. It is an observational window into HIV/AIDS treatment access pressure. Access to HIV/AIDS medicines is one of the most representative historical contexts of compulsory licensing practice. This study therefore uses ART coverage to test whether treatment coverage gaps are empirically associated with overall compulsory licensing mobilization. It does not generalize ART coverage as a proxy for all access to medicines or all public health pressure (41). Therefore, the study adds a supplementary HIV/AIDS-related compulsory licensing measure outside the main analysis to reduce disease-area mismatch between ART coverage and non-HIV/AIDS compulsory licensing events.

The log of AIDS deaths, ln_aids_deaths, and the TB incidence rate, tb_inc_rate, are used to measure disease burden pressure. They reflect pressure from HIV/AIDS disease outcomes and the burden of tuberculosis, respectively. Because AIDS deaths and TB incidence do not directly measure treatment coverage for medicines, this study includes them together with ART coverage to avoid conflating treatment access pressure with general disease burden. In other words, public health pressure is not reduced to a single ART indicator. ART coverage is used to observe treatment access pressure, while AIDS deaths and TB incidence are used to observe disease burden pressure.

Current health expenditure as a share of GDP, current_health_expenditure_pct_gdp, and out-of-pocket expenditure as a share of current health expenditure, oop_pct_current_health_expenditure, are used to measure health system absorptive conditions. They, respectively, reflect health system resource input and patients’ payment burden. They are not disease burden indicators and should not be simply classified as general economic structure variables. Rather, they are important background conditions for whether public health pressure can be institutionally absorbed.

The log of Global Fund HIV assistance, ln_gf_hiv, is used to measure the strength of a country’s connection to global health governance and procurement implementation networks. The variable is coded as ln (1 + gf_hiv_disbursed), where gf_hiv_disbursed is the annual country-level HIV disbursement recorded in Global Fund data. Because Global Fund disbursement data are record-based, country-years for which no HIV disbursement record is matched are interpreted as having no observed HIV disbursement in that year and are coded as 0. After the ln(1 + x) transformation, their value remains 0. This treatment retains country-years with no accepted or observed Global Fund HIV disbursement, but it also means that this study identifies observed Global Fund HIV funding connections rather than complete global health governance capacity.

General economic and structural controls include the log of GDP per capita, ln_gdp_pc; the log of total population, ln_pop; urbanization, urban_population_pct; and the share of manufactured exports in merchandize exports, manufactures_exports_pct_merchandise_exports. These variables control for economic development, country size, population structure, and general supply capacity. Economic development affects fiscal capacity and administrative resources, while manufactured exports reflect general supply capacity and trade structure (19, 20, 24, 36). However, these variables are only general capacity indicators and may not capture the specific administrative initiation, procurement coordination, and global health governance connection capacity required for compulsory licensing mobilization. If these conventional variables cannot stably explain compulsory licensing use, compulsory licensing mobilization cannot be reduced simply to economic development or industrial scale.

### 3.4 Panel design and main model

The main model uses a two-way fixed effects linear probability model, TWFE-LPM, specified as follows:

$$\begin{array}{l} \begin{array}{l} \mathrm{CL}\_\mathrm{it}=\mathrm{al}\mathrm{pha}\_i+\text{gamma}\_t+\text{beta}\_1^{*}\;\\ \mathrm{ART}\_\mathrm{it}+\text{beta}\_2^{*}\;\ln(\mathrm{GF}\_\mathrm{HIV})\_\mathrm{it}+\text{beta}\_3^{*}\; \end{array}\\ \ln(\mathrm{GDP}\_\mathrm{pc})\_\mathrm{it}+\text{beta}\_4^{*}\;X\_\mathrm{it}+\text{epsilon}\_\mathrm{it} \end{array}$$

Here, CL_it is the compulsory licensing event indicator for country i in year t. The terms alpha_i and gamma_t denote country and year fixed effects, respectively. ART_it is ART coverage, ln(GF_HIV)_it is the log of Global Fund HIV assistance, and ln(GDP_pc)_it is the log of GDP per capita. X_it is a vector of other control variables. X_it includes disease burden variables, health system absorptive conditions, and general structural controls. Disease burden variables include the log of AIDS deaths and TB incidence. Health system absorptive conditions include current health expenditure as a share of GDP and out-of-pocket expenditure as a share of current health expenditure. General structural controls include population size, urbanization, and the share of manufactured exports. Standard errors are clustered by country to account for within-country correlation.

LPM is selected rather than fixed effects logit as the main model because compulsory licensing events are infrequent, while LPM coefficients can be interpreted as probability changes and compared consistently across the main specifications. The one-period lag model uses explanatory variables at t-1 to predict compulsory licensing events at t, reducing simultaneity concerns. These models estimate observational country-year associations and should not be interpreted as causal identification.

### 3.5 Robustness and supplementary analyses

The model structure is organized in four layers. Table 1 reports the main TWFE-LPM analysis of compulsory licensing mobilization. Table 2 reports robustness checks using GEE logit models for repeated binary events, first-event models for first compulsory licensing mobilization, and Poisson models for event counts. Table 3 uses HIV/AIDS-related compulsory licensing events as a supplementary disease-area matching analysis. Table 4 uses ART coverage as the dependent variable to examine the boundary of outcome interpretation. These models serve different interpretive purposes and do not substitute for causal identification.

**Table 1:** Main TWFE-LPM analysis of compulsory licensing mobilization (WTO risk set, *N* = 1,690).

Variable	Any CL (current)	Any CL (lag 1)	Executed CL (current)	Executed CL (lag 1)	CL count (current)	CL count (lag 1)
ART coverage	−0.0017*** (0.0006)	−0.0014** (0.0007)	−0.0016*** (0.0005)	−0.0011* (0.0006)	−0.0025*** (0.0008)	−0.0021** (0.0010)
ln(GF HIV)	0.0019* (0.0010)	0.0028** (0.0012)	0.0013 (0.0009)	0.0023** (0.0012)	0.0027* (0.0015)	0.0038** (0.0018)
ln(GDP per capita)	0.0339 (0.0239)	0.0066 (0.0249)	0.0394* (0.0209)	0.0177 (0.0213)	0.0187 (0.0425)	−0.0062 (0.0389)
Manufactured exports share	−0.0001 (0.0005)	−0.0001 (0.0003)	0.0001 (0.0004)	0.0000 (0.0003)	−0.0001 (0.0005)	−0.0001 (0.0004)
Country fixed effects	Yes	Yes	Yes	Yes	Yes	Yes
Year fixed effects	Yes	Yes	Yes	Yes	Yes	Yes
Clustered standard errors	Country	Country	Country	Country	Country	Country

Country-clustered robust standard errors are in parentheses. *p < 0.1, **p < 0.05, ***p < 0.01. Control variables include ln(population), urbanization, current health expenditure as a share of GDP, out-of-pocket expenditure, TB incidence, and ln(AIDS deaths). They are not fully reported due to space limitations. Any CL, any compulsory licensing-related event; Executed CL, actually executed compulsory licensing event; CL count, count of compulsory licensing-related events; CL, compulsory licensing; ART, antiretroviral therapy; GF HIV, observed Global Fund HIV assistance; TWFE-LPM, two-way fixed effects linear probability model; ln, natural logarithm; GDP, gross domestic product; TB, tuberculosis.

**Table 2:** Robustness checks using GEE, first-event, and Poisson models.

Model	Dependent variable	ART coverage, B/p value	ln(GF HIV), B/*p* value
GEE logit, current	Any CL	−0.005/0.622	0.050/0.029**
GEE logit, lag 1	Any CL	−0.004/0.639	0.070/0.013**
GEE logit, current	Executed CL	−0.015/0.324	0.060/0.034**
GEE logit, lag 1	Executed CL	−0.008/0.642	0.083/0.017**
First-event logit, lag 1	First CL	0.021/0.150	0.083/0.014**
First-event cloglog, lag 1	First CL	0.020/0.133	0.071/0.033**
Poisson, current	CL count	−0.071/0.029**	0.044/0.289
Poisson, lag 1	CL count	−0.073/0.029**	0.057/0.194

These models are robustness checks. GEE models use an exchangeable correlation structure and year fixed effects. The first-event model risk set is *N* = 843, covering 79 countries and 42 first events. Poisson models include country and year fixed effects. Coefficients are reported on different model scales and should not be compared directly across model families. *p < 0.1, **p < 0.05, ***p < 0.01. GEE, generalized estimating equations; ART, antiretroviral therapy; B, coefficient estimate; *p*, *p* value; GF HIV, observed Global Fund HIV assistance; CL, compulsory licensing; ln, natural logarithm.

**Table 3:** Supplementary disease-area matching models for HIV/AIDS-related compulsory licensing.

Model	Dependent variable	ART coverage, B/*p* value	ln(GF HIV), B/p value	N/countries
TWFE-LPM	Any HIV/AIDS-related CL	−0.00102/0.093*	0.00076/0.418	1690/90
TWFE-LPM	Executed HIV/AIDS-related CL	−0.00131/0.010**	0.00098/0.228	1690/90
GEE logit	Any HIV/AIDS-related CL	0.00734/0.622	0.03027/0.222	1690/90
GEE logit	Executed HIV/AIDS-related CL	−0.00434/0.759	0.04804/0.102	1690/90
Poisson	HIV/AIDS-related CL count	−0.04662/0.160	0.00040/0.992	1690/90

These are supplementary disease-area matching models. HIV/AIDS-related compulsory licensing is measured using a strict disease-area matching definition. ART coverage is an HIV/AIDS treatment coverage indicator and does not represent all dimensions of pharmaceutical access. * *p* < 0.1, ** *p* < 0.05, *** *p* < 0.01. TWFE-LPM, two-way fixed effects linear probability model; GEE, generalized estimating equations; CL, compulsory licensing; ART, antiretroviral therapy; B, coefficient estimate; *p*, *p* value; GF HIV, observed Global Fund HIV assistance; ln, natural logarithm; N/countries, observations/countries.

**Table 4:** Supplementary outcome-boundary models for HIV/AIDS-related compulsory licensing and ART coverage.

Compulsory licensing variable	B	SE	*p*
Any HIV/AIDS-related CL, lag 1 year	−0.616	0.824	0.455
Any HIV/AIDS-related CL, lag 2 years	0.069	0.981	0.944
Any HIV/AIDS-related CL, lag 3 years	−0.533	0.764	0.485
Executed HIV/AIDS-related CL, lag 1 year	−0.949	0.874	0.278
HIV/AIDS-related CL post indicator	4.590	1.802	0.011
Any HIV/AIDS-related CL, lead 1 year	−1.168	0.773	0.131
Any HIV/AIDS-related CL, lead 2 years	−0.867	0.868	0.318
Executed HIV/AIDS-related CL, lead 1 year	−1.505	0.825	0.068

These are supplementary outcome-boundary models and do not estimate a strict causal policy effect. The dependent variable is ART coverage, an HIV/AIDS treatment coverage indicator that does not represent all dimensions of pharmaceutical access. All models include country and year fixed effects with country-clustered robust standard errors. Lead variables test whether future HIV/AIDS-related compulsory licensing predicts current ART coverage. ART, antiretroviral therapy; CL, compulsory licensing; B, coefficient estimate; SE, standard error; *p*, *p* value.

The supplementary analyses address two boundary questions. First, the study replaces all compulsory licensing events with HIV/AIDS-related compulsory licensing events to test whether the relationship between ART coverage and compulsory licensing mobilization remains after disease-area matching. Second, it uses ART coverage as the dependent variable to test whether HIV/AIDS-related compulsory licensing events are followed by observable changes in HIV/AIDS treatment coverage. These outcome-boundary models test one- to three-year lags, a post indicator after the first HIV/AIDS-related compulsory licensing event, and lead variables. Lead variables examine whether countries that later experience HIV/AIDS-related compulsory licensing were already on different ART coverage trajectories before the event.

## 4 Results

### 4.1 Sample description and descriptive statistics

After correction for WTO membership and accession year, the final risk set contains 2,276 country-year observations for 103 WTO members. Among them, 110 country-years, or 4.83 percent, record at least one compulsory licensing-related event, and 88 country-years, or 3.87 percent, record an executed compulsory licensing event. Of the 103 members, 59 experienced at least one compulsory licensing-related event during the observation period. Compulsory licensing events are temporally concentrated from 2004 to 2008, the early post-Doha period, and are concentrated by disease area in HIV/AIDS-related medicines. The low frequency and uneven distribution of compulsory licensing events are consistent with descriptions in the existing literature (6, 9) and support the interpretation of compulsory licensing as a conditionally mobilized instrument rather than a routine policy tool.

Under the strict disease-area matching measure, the MLP event table contains 114 HIV/AIDS-related compulsory licensing events. After entry into the WTO risk set, there are 95 events involving 83 country-years and 52 countries. This shows that HIV/AIDS is indeed an important historical context of compulsory licensing practice, but all compulsory licensing events still include other disease and drug areas. Interpretive boundaries must therefore be maintained between the main analysis and the supplementary HIV/AIDS-related analysis. Table 5 reports the descriptive statistics for the main variables.

**Table 5:** Descriptive statistics for main variables (WTO risk set, *N* = 2,276).

Variable	Valid N	Mean	SD	Min.	Max.
Any compulsory licensing event	2,276	0.048	0.215	0	1
Executed compulsory licensing event	2,276	0.039	0.193	0	1
Number of compulsory licensing events	2,276	0.058	0.299	0	5
ART coverage (%)	2028	32.41	26.16	0.00	95.56
Global Fund HIV assistance (million USD)	1,418	15.70	25.23	0.15	179.35
ln(Global Fund HIV)	2,276	9.76	7.67	0.00	19.00
GDP per capita (USD)	2,264	3366.71	3052.71	111.41	15892.72
Health expenditure as % of GDP	2,163	5.47	2.31	1.33	23.09
Out-of-pocket expenditure as % of health expenditure	2,163	39.35	19.56	2.17	87.90
Manufactured exports share (%)	1982	35.81	29.50	0.00	372.92
TB incidence (per 100,000)	2,276	214.61	251.75	0.00	2650.00
Population (million)	2,276	55.93	191.67	0.07	1450.94
Urbanization rate (%)	2,276	47.63	19.40	8.24	92.95

SD, standard deviation; Min., minimum; Max., maximum; ART, antiretroviral therapy; GDP, gross domestic product; HIV, human immunodeficiency virus; TB, tuberculosis; USD, United States dollars; ln, natural logarithm; GF HIV, Global Fund HIV assistance; CL, compulsory licensing.

Because variables such as ART coverage, Global Fund HIV assistance, health expenditure, and manufactured exports have varying degrees of missingness, the regression models use complete-case analysis. The effective sample entering the TWFE-LPM main models is 1,690 country-years, covering 90 countries. The risk set for the first-event models contains 843 country-years, covering 79 countries. Observations not entering the models do not contribute to coefficient estimation. The results should therefore be interpreted as observational associations based on the complete-case sample. Missing data may introduce sample selection risks, which are discussed in the limitations section.

Figure 1 summarizes the annual distribution of compulsory licensing events in the WTO risk set.

**Figure 1:**
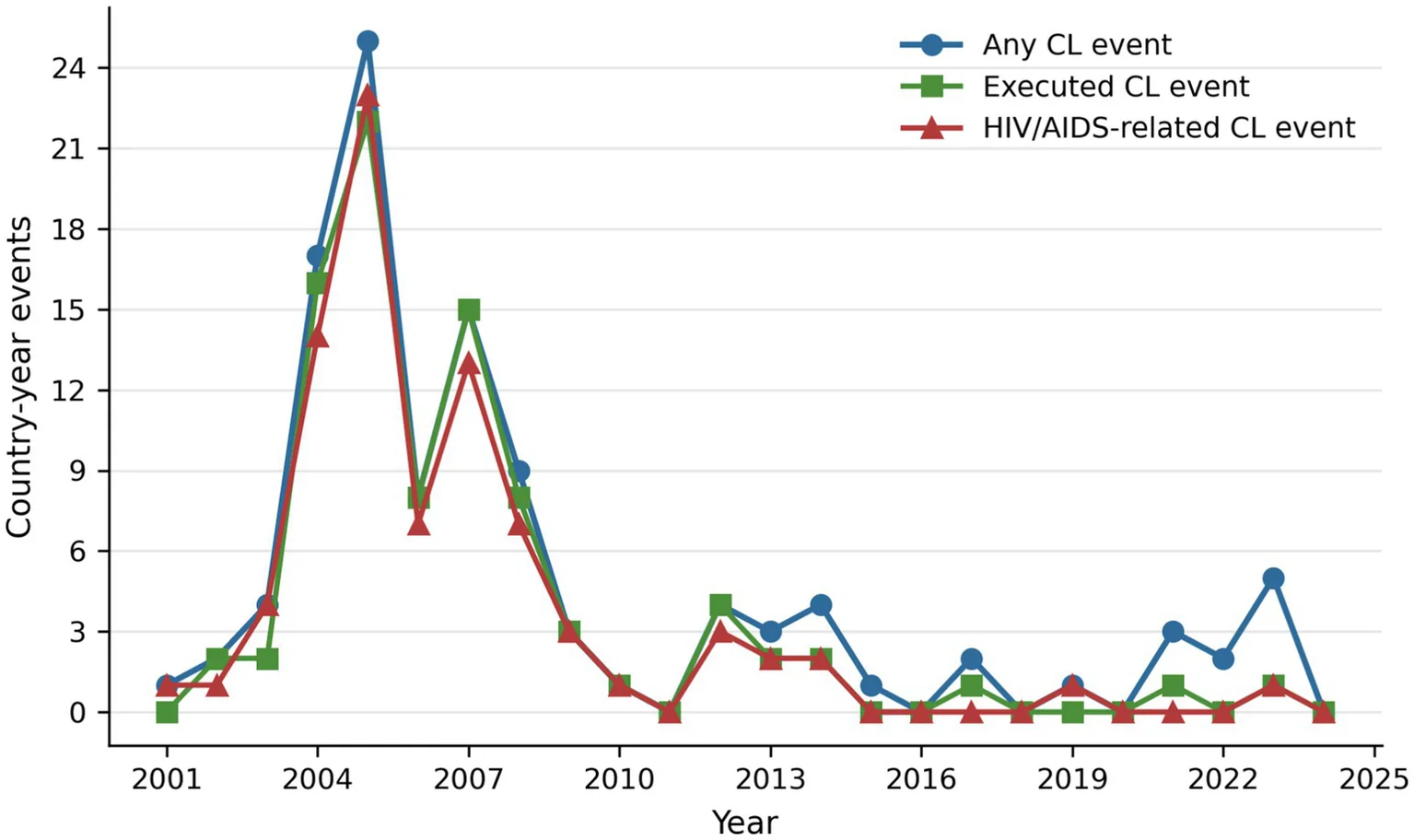
Annual distribution of compulsory licensing events in the WTO risk set.

### 4.2 Main TWFE-LPM results

Table 1 reports the main TWFE-LPM results for the WTO risk set. In the main models, ART coverage is negatively associated with all three compulsory licensing measures. The association is statistically significant for current ART coverage in models of any compulsory licensing event, executed compulsory licensing events, and event count, and it remains negative in the one-period lag specifications. Interpreted within the LPM framework, lower ART coverage is associated with a higher probability or count of compulsory licensing-related events. These results support the view that HIV/AIDS treatment access pressure is associated with compulsory licensing mobilization in the main country-year models, while they do not establish causal effects.

For disease burden variables, AIDS deaths and TB incidence are included in all main models to observe pressure from HIV/AIDS disease outcomes and tuberculosis burden. In current-value TWFE models, neither AIDS deaths nor TB incidence reaches conventional significance for any compulsory licensing event, executed compulsory licensing event, or event count. RQ1 is therefore partially supported: treatment access pressure better explains compulsory licensing mobilization in the main TWFE models, while disease burden variables do not show independent and cross-model consistent explanatory power.

Global Fund HIV assistance is positively associated with compulsory licensing under the all-event measure in the main TWFE-LPM models, with clearer support in the one-period lag specifications. This pattern should be interpreted as an association between observed Global Fund HIV funding connections and institutional mobilization, not as evidence that the Global Fund directly causes compulsory licensing events. The result is assessed further in the robustness and disease-area matching analyses.

GDP per capita does not reach conventional significance in either current-value or one-period lag models. Current health expenditure as a share of GDP and out-of-pocket expenditure likewise do not show stable significant relationships. The share of manufactured exports may show marginal results in some other samples or models, but it is not significant in the TWFE main models for the 103-country WTO risk set and lacks cross-model stability. These results indicate that compulsory licensing mobilization cannot be reduced simply to economic development, the scale of health spending, or general manufacturing export capacity.

Figure 2 visualizes the core coefficient estimates from the main TWFE-LPM models.

**Figure 2:**
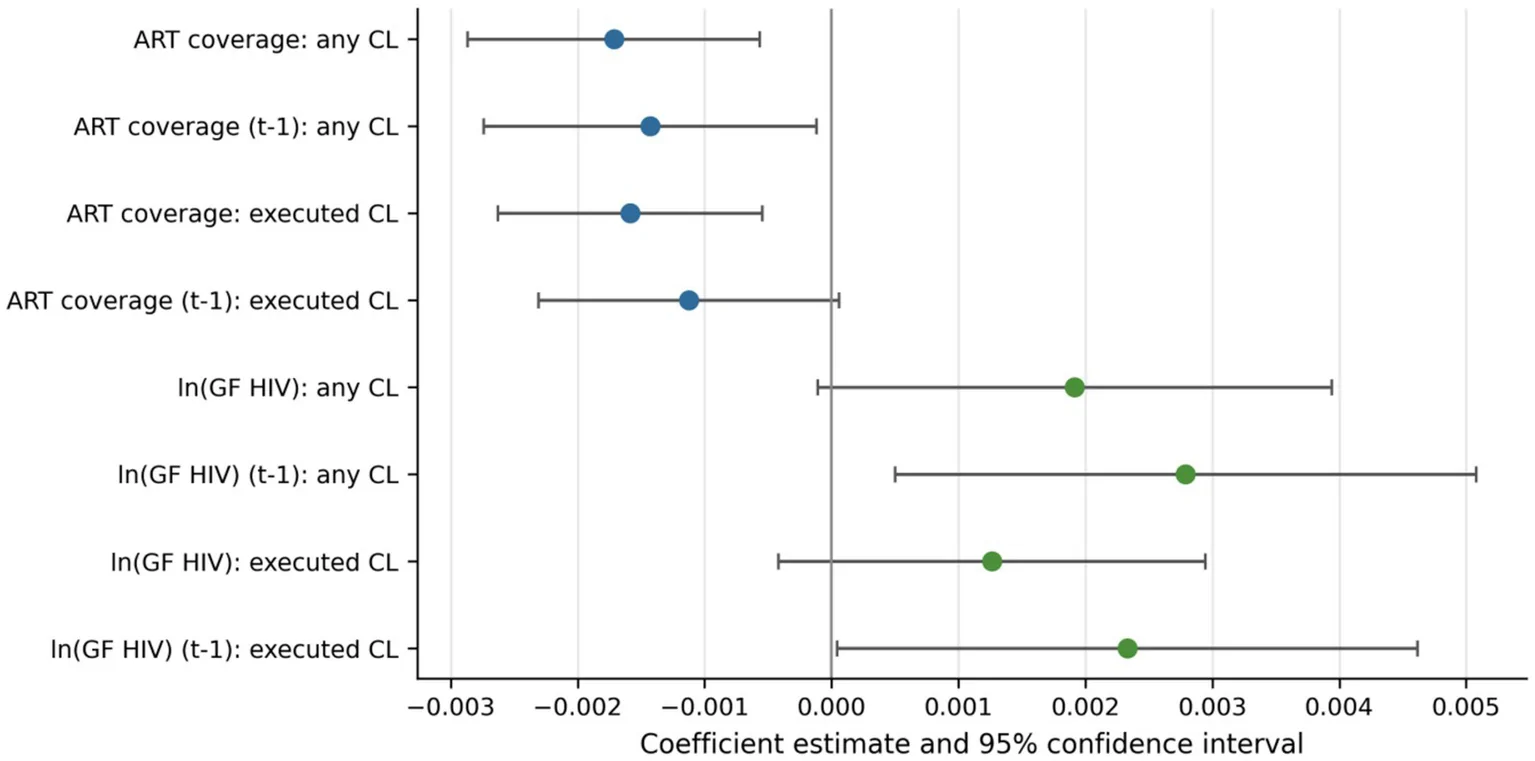
Core coefficients in the TWFE-LPM main models.

### 4.3 Robustness checks: GEE, first-event, and Poisson models

Table 2 reports robustness checks using model families that answer different questions from the main TWFE-LPM models. In GEE logit models, Global Fund HIV assistance is positively associated with any compulsory licensing and executed compulsory licensing in both current and lagged specifications. ART coverage does not reach conventional significance in the GEE models, which indicates that the ART result is sensitive to model family.

The discrete-time first-event models focus on the risk of a first compulsory licensing event rather than repeated mobilization. In these models, one-period lagged Global Fund HIV assistance is positively associated with first-event risk in both logit and cloglog specifications. ART coverage does not show the same support in first-event models, suggesting that the conditions associated with first mobilization and repeated event occurrence may differ.

In Poisson count models, ART coverage remains negatively associated with compulsory licensing event counts in both current-value and one-period lag models. Global Fund HIV assistance does not reach conventional significance in the Poisson models, although the direction is positive. Taken together, the robustness checks support a directional but not fully cross-model stable pattern: treatment access pressure is clearer in the main TWFE-LPM and Poisson count models, while observed Global Fund HIV assistance is clearer in the GEE and first-event binary models.

### 4.4 Supplementary disease-area matching: HIV/AIDS-related compulsory licensing

ART coverage reflects HIV/AIDS treatment access pressure and cannot represent access to medicines in all disease areas. The supplementary disease-area matching analysis therefore replaces all compulsory licensing events with HIV/AIDS-related compulsory licensing events.

Table 3 shows that the direction of the ART coverage association remains negative after disease-area matching. In TWFE-LPM models, ART coverage is marginally negatively associated with any HIV/AIDS-related compulsory licensing event and significantly negatively associated with executed HIV/AIDS-related compulsory licensing events. This supports the interpretation that the relationship between ART coverage and compulsory licensing mobilization is not entirely driven by the inclusion of non-HIV/AIDS drug events in the all-event measure.

The supplementary HIV/AIDS-related models also define the interpretive boundary. ART coverage does not reach conventional significance in GEE logit models or in the Poisson count model. Global Fund HIV assistance is generally positive under the HIV/AIDS-related measure, but it does not form a stable significant relationship. The Global Fund result is therefore better interpreted as an observed resource-connection condition for overall institutional mobilization than as an independent cause of compulsory licensing events in a single disease area.

Figure 3 reports the core coefficients under the supplementary HIV/AIDS-related compulsory licensing measure.

**Figure 3:**
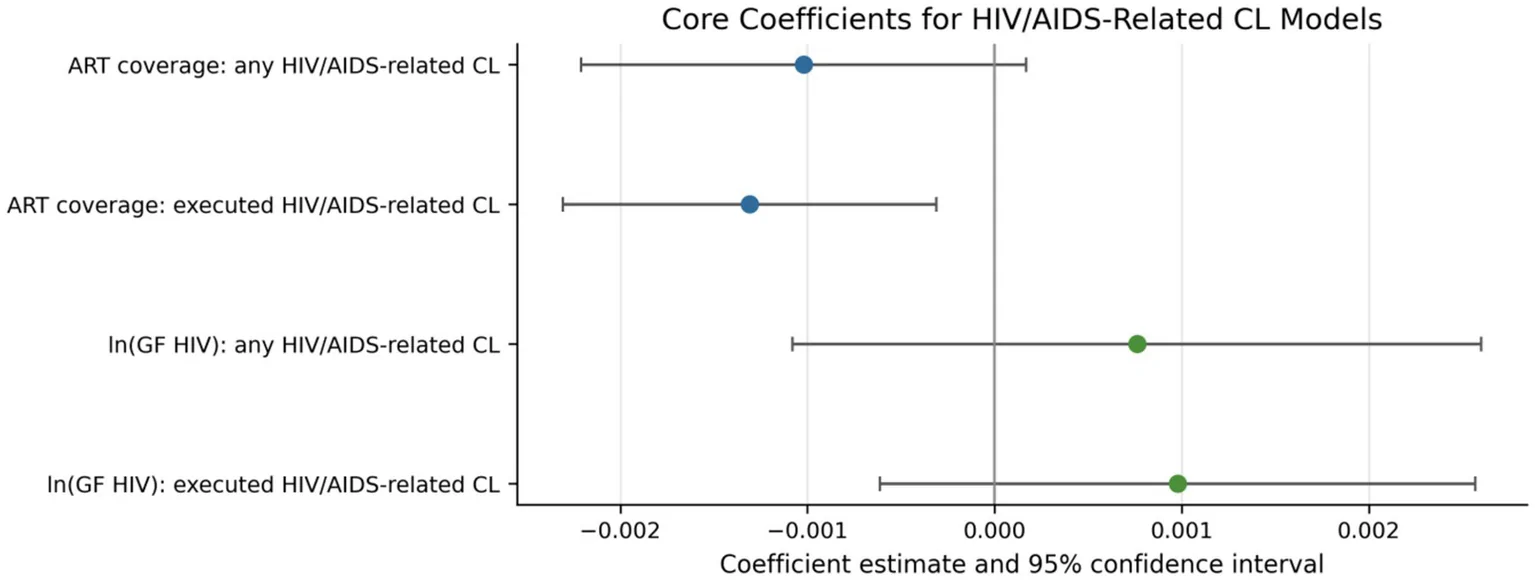
Core variable coefficients under the Supplementary HIV/AIDS-related compulsory licensing measure.

### 4.5 Supplementary outcome-boundary models: HIV/AIDS-related compulsory licensing and changes in ART coverage

To distinguish institutional mobilization from health outcome conversion, this study uses ART coverage as the dependent variable and tests whether HIV/AIDS-related compulsory licensing events are associated with subsequent changes in HIV/AIDS treatment coverage. Unlike the preceding mobilization models, this section examines the boundary of outcome interpretation rather than estimating a strict policy effect.

Table 4 shows that the lagged terms of HIV/AIDS-related compulsory licensing do not support the strong causal claim that compulsory licensing improves ART coverage. One-year, two-year, and three-year lagged measures of any HIV/AIDS-related compulsory licensing do not reach conventional significance, and one-year lagged executed HIV/AIDS-related compulsory licensing is also not significant. Current cross-national country-year data therefore do not support interpreting HIV/AIDS-related compulsory licensing use as a direct cause of improved ART coverage.

The post_hiv_any variable is positive and significant, indicating that countries that experienced HIV/AIDS-related compulsory licensing have higher ART coverage in the years after compulsory licensing. This result should not be interpreted as showing that compulsory licensing caused higher ART coverage. The lag models do not significantly predict ART coverage improvement, and the observational panel cannot exclude alternative explanations such as treatment policy expansion, international assistance, health system absorptive capacity, and long-term within-country trends.

Figure 4 presents the outcome boundary models for HIV/AIDS-related compulsory licensing and ART coverage.

**Figure 4:**
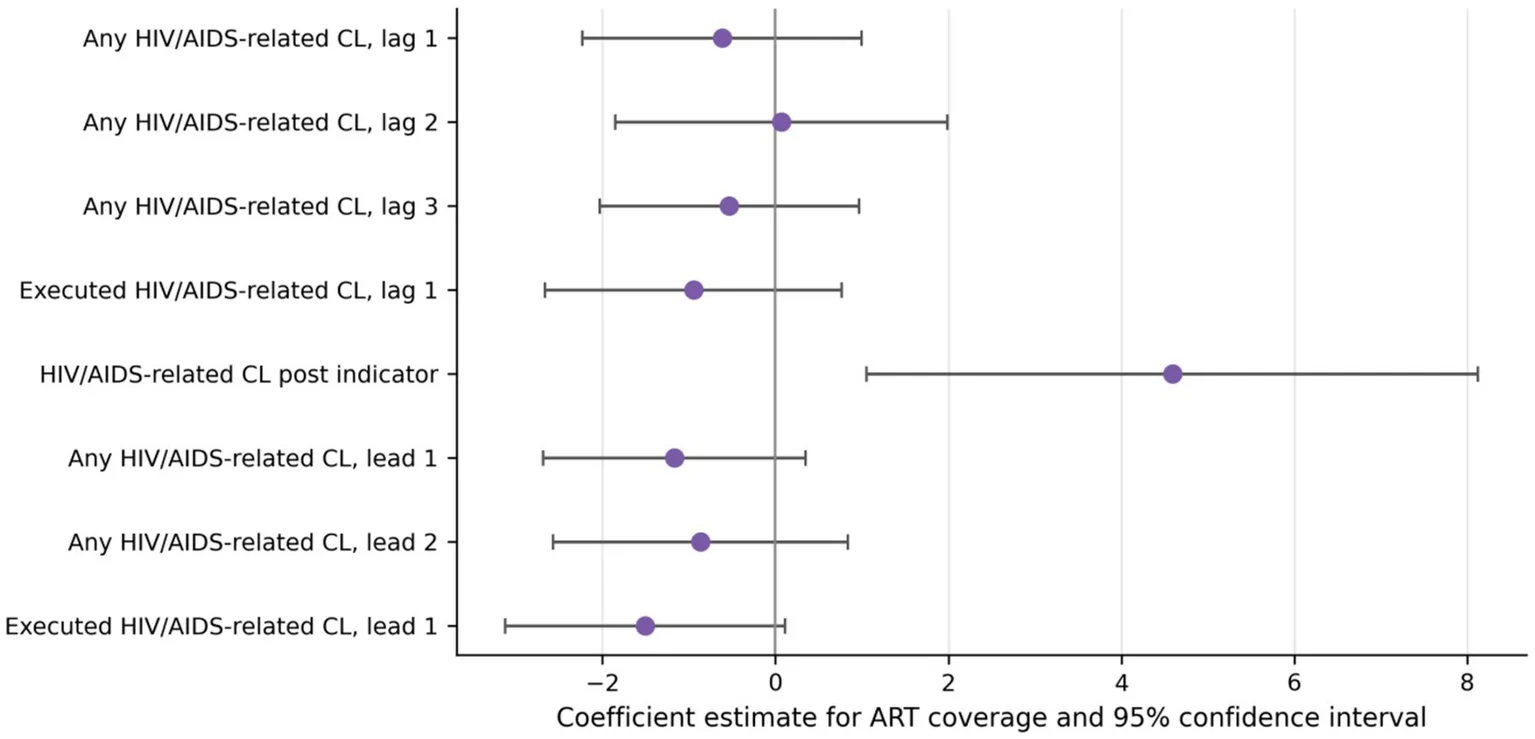
Outcome boundary models for HIV/AIDS-related compulsory licensing and ART coverage.

### 4.6 Summary of results

The analyses above yield five main findings. First, lower ART coverage is negatively associated with the occurrence and count of all compulsory licensing events in the main TWFE-LPM models and in Poisson count models, although the result is not stable across all model families. This supports a cautious association between HIV/AIDS treatment access pressure and compulsory licensing mobilization.

Second, after disease-area matching, ART coverage remains negatively associated with HIV/AIDS-related compulsory licensing and is significant for executed HIV/AIDS-related compulsory licensing. This indicates that the ART association is not entirely driven by the inclusion of non-HIV/AIDS drug compulsory licensing events.

Third, AIDS deaths and TB incidence, as disease burden variables, do not form stable main effects across models. The conclusion about public health pressure should therefore be narrowed. This study more strongly supports an empirical link between treatment access pressure and compulsory licensing mobilization. It does not support interpreting disease burden pressure itself as a stable main effect in the initiation of compulsory licensing.

Fourth, observed Global Fund HIV assistance is positively associated under the all-compulsory-licensing measure and is supported in GEE and first-event models, but it does not form stable significant results under the supplementary HIV/AIDS-related compulsory licensing measure. This suggests that Global Fund HIV assistance is better interpreted as an observed governance-resource connection for overall institutional mobilization than as a direct cause of compulsory licensing events in a single disease area or as a complete measure of global health governance capacity.

Fifth, economic structure and health system absorptive variables, including GDP per capita, health expenditure, out-of-pocket expenditure, and the share of manufactured exports, do not constitute stable main effects. The lagged terms of HIV/AIDS-related compulsory licensing also do not support the strong causal claim that compulsory licensing improves ART coverage. Overall, compulsory licensing-related events are associated in some models with HIV/AIDS treatment access pressure and observed governance-resource connections, but their use does not necessarily appear in cross-national data as a directly observable improvement in treatment coverage.

## 5 Discussion

The significance of these results is not that compulsory licensing is simply related to public health pressure. Rather, the results clarify the conditional structure of actual compulsory licensing mobilization within pharmaceutical access governance. The existence of legal space (2, 5, 11, 14–16) does not automatically produce actual use or improve treatment coverage. Within a country-year risk set corrected for WTO membership and accession year, actual compulsory licensing mobilization cannot be explained simply as the natural result of legal authorization, general disease burden, or state capacity. It is more clearly associated in this study with gaps in HIV/AIDS treatment coverage and observed governance-resource connections. This interpretation links international economic law to public health policy by showing that legal flexibility matters for medical security systems only when it can be absorbed through administrative action, procurement arrangements, and health-governance resources.

### 5.1 Main findings and the institutional mobilization explanation

The negative result for ART coverage suggests that compulsory licensing is better positioned as a policy response instrument mobilized under pressure from HIV/AIDS treatment access, rather than as a routine policy instrument that members naturally activate once they possess legal space. When HIV/AIDS treatment coverage is insufficient, governments may face greater public health accountability pressure, drug supply gaps, and price negotiation pressure, which may increase policy demand to mobilize compulsory licensing to expand generic supply or strengthen bargaining leverage. This finding is consistent with case studies of compulsory licensing in Thailand, Brazil, and other countries (7, 8, 21, 22, 48), and it also echoes research on ARV prices and the bargaining function of compulsory licensing (10, 37). At the same time, ART coverage is relatively stable as an indicator of HIV/AIDS treatment access pressure, while AIDS deaths and TB incidence do not form stable main effects across models. This pattern suggests that compulsory licensing mobilization is more closely associated with gaps in drug treatment coverage and price access pressure than with a mechanical response to general disease severity.

To reduce disease-area mismatch between ART coverage and non-HIV/AIDS drug compulsory licensing events, this study further uses the supplementary HIV/AIDS-related compulsory licensing measure. The results still show a significant negative relationship between ART coverage and executed HIV/AIDS-related compulsory licensing, indicating that the main result is not entirely caused by the inclusion of non-HIV/AIDS drug events. However, ART coverage does not reach significance in GEE and first-event models, suggesting that the mechanisms underlying first compulsory licensing initiation and different model specifications may be more complex and cannot be fully explained by a single treatment access indicator.

The positive result for Global Fund HIV assistance is an important contribution of this study relative to previous research, but its interpretation must remain limited to the observed variable. Existing compulsory licensing research has focused more on treaty interpretation, domestic legislative adoption, price negotiation function, external trade pressure, and institutional barriers (4, 10–12, 17, 18, 21, 22, 24, 26, 27, 37). It has less often treated connections to global health governance resources as a cross-national empirical variable associated with actual compulsory licensing mobilization. At the same time, global health governance research usually discusses the Global Fund, pooled procurement, and supply chain mechanisms as public health governance tools that improve drug prices, supply stability, and treatment coverage (29–33, 35). It seldom examines them within the framework of institutional mobilization for actual use of TRIPS compulsory licensing. This study introduces Global Fund HIV assistance into a country-year panel analysis not as a single cause that directly triggers compulsory licensing, but as a proxy for observed funding and governance-resource connections. The positive association in the all-event main models and several robustness models may suggest that actual compulsory licensing mobilization is linked not only to public health pressure itself but also to external resource connections that can support the conversion of legal space into policy action.

This finding also moves compulsory licensing research beyond explanations based only on domestic legal space and state capacity toward the question of how global health governance networks are associated with the practical availability of policy space in international economic law. Existing research has shown that compulsory licensing has public health purposes, legal legitimacy, and price negotiation functions, and has also identified administrative procedures, supply capacity, and political pressure as barriers to initiation (10, 14, 17, 18, 24, 37). The Global Fund provides not only financial support but also governance connections with recipient health systems through project management, procurement rules, performance assessment, and accountability mechanisms (29–31). Research on procurement and supply chains also shows that pooled procurement, market shaping, and international supply networks may affect drug prices, supply stability, and treatment access (32, 33, 35). The positive result for Global Fund HIV assistance cannot be interpreted as the Global Fund directly promoting compulsory licensing initiation, nor can it be understood as a measure of the full scope of global health governance capacity. More precisely, it captures observed HIV funding and governance-resource connections. The results suggest that these observed connections may be one condition associated with the feasibility of compulsory licensing mobilization, especially when public health pressure already exists.

GDP per capita, health expenditure, out-of-pocket expenditure, and manufactured exports do not show results stable enough to support the main conclusions across models. This makes it difficult to reduce compulsory licensing mobilization to general wealth, the scale of health spending, patients’ payment pressure, or industrial scale. Existing research has noted that compulsory licensing initiation faces multiple barriers, including administrative procedures, compensation arrangements, procurement coordination, supply organization, pharmaceutical capacity, and external political pressure (14, 17–20, 24). The results of this study are consistent with that judgment, while indicating that these barriers are not captured well by macroeconomic development or general health investment alone. Actual use of compulsory licensing may be more closely associated with specific administrative initiation capacity, procurement organization capacity, supply arrangements, and global health governance cooperation capacity.

The findings from the ART coverage outcome models further show that the institutional mobilization conditions of compulsory licensing cannot be equated directly with health outcome conversion. One- to three-year lags of HIV/AIDS-related compulsory licensing do not significantly predict increases in ART coverage, indicating that current cross-national country-year data do not support interpreting compulsory licensing use as a direct cause of improved ART coverage. This finding does not deny the institutional value of compulsory licensing. Existing research has shown that compulsory licensing may affect access to medicines indirectly through bargaining leverage, expansion of procurement channels, signals for generic market entry, and policy demonstration effects (10, 28, 35, 37, 38). These pathways, however, are difficult to observe directly in country-year cross-national panels. The conversion of compulsory licensing from legal authorization to treatment coverage improvement requires several steps, including drug registration, supplier organization, procurement contracts, fiscal payment, and health service system absorption. Insufficiency at any step may prevent compulsory licensing use from appearing in the short term as an increase in ART coverage. This study therefore understands compulsory licensing as a legal and policy instrument associated with treatment access pressure, rather than as a health outcome improvement mechanism that current cross-national data can directly prove.

Taken together, these results show that the key issue in TRIPS public health exceptions is not only whether the treaty provides policy space, but whether that policy space can be absorbed by domestic administrative action, drug procurement systems, and transnational public health governance resources. Park and Konken’s research on domestic legislative adoption explains how the legal space of compulsory licensing enters members’ domestic law (11). Son’s research on the time structure of attempted compulsory licensing and intellectual property institutions shows that compulsory licensing does not occur automatically after treaty authorization (12, 26). Park’s research on democratic institutions and regulatory threats further demonstrates the political economy conditions of compulsory licensing (27). Building on these studies, this article advances the object of analysis to actual use events and simultaneously incorporates treatment access pressure, disease burden, health system absorptive conditions, and connections to global health governance resources into a risk set corrected for WTO membership. The contribution of this study is not to repeat that compulsory licensing has a public health purpose, but to reveal that an empirical gap remains between public health purpose, legal authorization, and actual institutional mobilization.

### 5.2 Policy implications

The results have implications for policy discussions on improving the practical availability of TRIPS compulsory licensing. Increasing practical availability may depend not only on revising legal texts or confirming treaty authorization, but also on reducing the institutional conversion costs between legal space and actual policy action. Existing research has pointed out that compulsory licensing initiation is constrained by administrative procedures, compensation arrangements, drug registration, procurement coordination, supplier organization, and the procedural costs of cross-border supply under Article 31bis (5, 14, 17, 18, 24). Members may therefore need institution-building around administrative initiation capacity and procurement coordination capacity. Clearer mechanisms for patent status identification, compensation assessment, licensing decisions, and linkage with drug regulation may help members complete administrative initiation more quickly when public health pressure emerges. Medicine supply, quality regulation, market access, procurement contracts, and fiscal payment after compulsory licensing authorization also need to be linked so that legal authorization does not remain merely formal. For medical security systems, these links shape whether pharmaceutical access governance can move from institutional reserve to treatment coverage and supply security.

A further policy implication is that the practical availability of TRIPS public health exceptions should not be understood only from the perspectives of domestic law and national administrative capacity. It should also be evaluated within networks of global health governance resources. The results show a positive association between observed Global Fund HIV assistance and compulsory licensing mobilization in the all-event main models and several robustness checks. Existing research on Global Fund financing, procurement rules, supply chain coordination, and governance accountability mechanisms provides the context for this interpretation (29–33, 35). International financing platforms, procurement alliances, and technical assistance networks may not only support access to medicines governance. They may also become absorptive conditions for members to mobilize policy space under international economic law. Increasing the usability of compulsory licensing is therefore not only a matter of making the law allow it. It also requires attention to whether members can connect legal authorization with financing, procurement, supply chain, information, and organizational implementation resources when public health pressure emerges.

This is especially important for lower- and middle-income members. For members lacking pharmaceutical manufacturing capacity, administrative resources, and procurement bargaining power, initiating compulsory licensing alone may still involve high implementation costs even where domestic law contains compulsory licensing provisions. For members without pharmaceutical manufacturing capacity, the Article 31bis cross-border supply route may require further simplification of notification, evidentiary, and procurement coordination requirements to reduce the cost of institutional mobilization (5, 14). Procedural simplification alone, however, may be insufficient. International organizations, global health funds, regional procurement platforms, and technical assistance networks may support patent information identification, supplier matching, drug registration, quality certification, and procurement implementation before and after compulsory licensing initiation. Reform of TRIPS public health exceptions should therefore be connected with implementation infrastructure linking domestic administrative agencies, international procurement platforms, and public health financing mechanisms.

### 5.3 Supplementary illustration from the Chinese experience

China provides a supplementary illustration of the institutional mobilization explanation rather than an additional cross-national empirical test. China has established a compulsory licensing system for pharmaceutical patents in domestic law, but there is no public record of formal use to date (49). This pattern should be understood within a broader access-to-medicines governance system in which national medical insurance negotiations, centralized procurement, and other administrative pricing and payment tools play a prominent role (50, 51). The Chinese experience therefore supports the narrower point developed in this study: the institutional value of compulsory licensing cannot be measured only by formal execution frequency. Where governments possess alternative administrative bargaining and procurement tools, compulsory licensing may remain a legal reserve or regulatory deterrent rather than being formally mobilized. This supplementary illustration is consistent with the cross-national finding that legal availability does not automatically become policy mobilization.

### 5.4 Limitations and future directions

The results should be understood as observational country-year associations, not strict causal identification. Several limitations should be stated clearly. First, the MLP database records compulsory licensing-related events observed in public sources. Zero values mean that no event was observed, not that an official authority confirmed non-occurrence. Some compulsory licensing events may therefore be omitted. Second, ART coverage reflects only the HIV/AIDS treatment access dimension of public health pressure and cannot represent all access to medicines needs. AIDS deaths and TB incidence can reflect the disease burden, but they do not directly measure gaps in drug treatment coverage and cannot substitute for treatment access pressure indicators. Third, Global Fund HIV assistance is a proxy for observed connection to global health governance resources. It cannot be equated with governance capacity itself or with the full scope of global health governance, and the interaction mechanisms between recipient countries and the Global Fund are difficult to capture fully in panel data. In addition, the Global Fund HIV assistance variable codes country-years with no matched HIV disbursement record as 0. If the original database has unrecorded disbursements or matching omissions, zero-value misclassification may occur. Related results should therefore be interpreted as associations between observed Global Fund HIV funding connections and compulsory licensing mobilization. Fourth, the main models use complete-case analysis, reducing the sample from 2,276 country-years in the WTO risk set to 1,690 country-years. If missingness is not random, for example, if countries with weaker data capacity or specific high public health pressures are more likely to lack key variables, the estimates may be affected by sample selection. Fifth, low-frequency events limit statistical power, especially in first-event models, analyses of non-executed compulsory licensing, and supplementary HIV/AIDS-related compulsory licensing models. Sixth, TWFE-LPM, GEE, first-event, and Poisson count models provide robustness checks from several angles. However, all of these models remain within an observational research framework. They cannot exclude the influence of unobserved confounders, such as changes in domestic political will, specific drug patent status, advocacy by international non-governmental organizations, and particular drug procurement negotiations.

Despite these limitations, this study contributes systematic cross-national observational evidence on the institutional mobilization conditions of compulsory licensing-related events based on a country-year risk set corrected for WTO membership. It also incorporates treatment access pressure and connections to global health governance resources into an empirical analysis of the practical availability of policy space in international economic law. Future research can advance causal identification through finer-grained event data, drug-level price and supply information, and natural experimental designs.

## 6 Conclusion

Based on 2,276 country-year observations for 103 WTO members, this study examines the public health conditions and governance-resource connections associated with the actual use of TRIPS compulsory licensing as a medicine access and medical security policy instrument. The results show that lower ART coverage, understood only as an HIV/AIDS treatment access indicator, is negatively associated with the occurrence of compulsory licensing-related events in the TWFE and Poisson models. Observed Global Fund HIV assistance is positively associated under the all-event measure and is supported in some robustness models. Supplementary analyses of HIV/AIDS-related compulsory licensing further show that, after disease-area matching, ART coverage remains significantly and negatively associated with executed HIV/AIDS-related compulsory licensing. This indicates that the empirical link between HIV/AIDS treatment access pressure and compulsory licensing mobilization is not entirely caused by the inclusion of non-HIV/AIDS drug compulsory licensing events. At the same time, AIDS deaths, TB incidence, GDP per capita, health expenditure, out-of-pocket expenditure, and the share of manufactured exports do not constitute stable main effects across models.

Supplementary outcome-boundary models do not support the strong causal claim that HIV/AIDS-related compulsory licensing events significantly improve ART coverage. This study therefore argues that TRIPS compulsory licensing should be understood as a legal and health policy instrument associated with treatment access pressure and observed governance-resource connections, rather than as a health outcome improvement mechanism that current cross-national data can directly prove. ART coverage should not be read as a general measure of pharmaceutical access, and Global Fund HIV assistance should not be read as the full scope of global health governance capacity. The public health significance of compulsory licensing may depend not only on whether treaty rules authorize an exception but also on whether members can convert exception rules into policy action that can be executed, procured, supplied, and coordinated. These findings provide empirical reference for assessing the practical availability of policy space in international economic law and for understanding the match between legal instruments, administrative capacity, and medicine access security in global public health governance. Future research should further examine changes in drug prices, supply, and market structure after compulsory licensing use, so as to complement the causal chain from institutional mobilization conditions to health outcome improvement.

## Data Availability

The study used publicly available data from the Medicines Law & Policy TRIPS Flexibilities Database, UNAIDS AIDSinfo, the Global Fund Data Service, the World Bank World Development Indicators, and WHO data platforms. Direct links: https://tripsflex-api.medicineslawandpolicy.org/flex, https://aidsinfo.unaids.org/public/documents/Estimates_2025_en.zip, https://data.api.theglobalfund.org/openapi.json, https://extranet.who.int/tme/generateCSV.asp?ds=estimates, https://ghoapi.azureedge.net/api/, and https://databank.worldbank.org/source/world-development-indicators.
